# Supplementary material for: Post-surgery spontaneous pneumothorax: Long-term recurrence rates and follow-up challenges revealed by a written survey
Source: PLoS One. 2024 Oct 10;19(10):e0307910. doi: 10.1371/journal.pone.0307910 (PMC11466418; doi:10.1371/journal.pone.0307910)
Supplement: S1 File — (DOCX) [file pone.0307910.s001.docx]

Survey Content

Question 1:

Since your surgery, have you experienced symptoms such as chest pain, back pain, or shortness of breath?

→ (Yes / No) Please circle one. If "No," the survey is complete.

Question 2:

For those who answered "Yes" to Question 1:

After the onset of symptoms, did you visit a medical facility other than our hospital?

→ (Yes / No) Please circle one. If "No," the survey is complete.

Question 3:

For those who answered "Yes" to Question 2:

Did you undergo imaging tests such as chest X-rays or chest CT scans at the medical facility, and were you diagnosed with "pneumothorax" or told that your lung had collapsed?

→ (Yes / No) Please circle one. If "Yes," which side was it? (Right / Left)
