# Supplementary material for: Post-surgery spontaneous pneumothorax: Long-term recurrence rates and follow-up challenges revealed by a written survey
Source: PLoS One. 2024 Oct 10;19(10):e0307910. doi: 10.1371/journal.pone.0307910 (PMC11466418; doi:10.1371/journal.pone.0307910)
Supplement: Attachment — Submitted filename: To reviewer.docx [file pone.0307910.s002.docx]

Comments to Reviewers

Thank you for reviewing my paper. I am pleased to see from your comments that you have read my paper in depth.

To Dr. Alessio Campisi:

I have corrected your comments regarding the proofreading of the text. I have added the questionnaire as additional information. I have also mentioned the treatment and prevention of recurrence of spontaneous pneumothorax in the discussion. Thank you very much for your evaluation.

To Dr. Yekta Altemur Karamustafaoglu:

I would like to emphasize that postoperative follow-up is more difficult in young patients with spontaneous pneumothorax. Therefore, I have included cases that did not respond to the survey. The proportion of patients who could not be followed up was higher among younger patients because their addresses and telephone numbers had changed since the time of treatment, preventing a prognostic survey.

Among the cases that responded to the survey, the ipsilateral recurrence rate in patients in their 50s and the contralateral incidence rate in patients in their 30s and 60s were 0%. It is possible that some non-responders were postoperative pneumothorax cases, but I suspect the number is small. I believe that growth and development are associated with the development of pneumothorax. The absence of pneumothorax in patients in their thirties or later may be due to the cessation of growth and development.

We did not use adhesion therapy for the initial treatment of all patients in our study. Although adhesion therapy may contribute to a decrease in the recurrence rate, we believe it is highly invasive due to the strong inflammatory effects. Particularly in young patients, we choose methods that do not induce adhesion (e.g., use of anti-adhesion agents) as much as possible. We have experienced cases of recurrence of neo-bulla in S1+2c, S3, and S6 in young patients. We believe this is because the same area was not cared for during the initial surgery. We believe that care, such as covering, is necessary even if no bulla is observed in some areas.

Thank you very much for your evaluation.
